# Prognostic nutritional index at admission predicts 90-day mortality in patients aged ≥80 years with hip fracture: a system-level readout of the nutrition–immune milieu relevant to microenvironment-responsive bone repair

**DOI:** 10.3389/fmed.2026.1788594

**Published:** 2026-04-17

**Authors:** Delong Yin, Sheng Lu, Shikai Li, Xinyue Cheng, Zhenbo Fan, Yuan Ye, Guoqing Jin

**Affiliations:** 1 Center of Orthopedics and Sports Medicine, Heyou Hospital, Foshan City, Guangdong, China; 2 Department of Orthopedics, ShiPai Hospital of Dongguan, Dongguan, Guangdong, China; 3 Department of Orthopedics, The Third Affiliated Hospital, Guangzhou Medical University, Guangzhou, Guangdong, China; 4 Guangdong Provincial Key Laboratory of Major Obstetric Diseases, Guangdong Provincial Clinical Research Center for Obstetrics and Gynecology, The Third Affiliated Hospital, Guangzhou Medical University, Guangzhou, China; 5 Department of Critical Care Medicine, Shenzhen Nanshan People’s Hospital, Shenzhen, China; 6 Department of Critical Care Medicine, The Third Affiliated Hospital, Guangzhou Medical University, Guangzhou, Guangdong, China

**Keywords:** frailty, hip fracture, malnutrition, mortality, prognostic nutritional index, super-elderly

## Abstract

**Background:**

Hip fractures in super-elderly patients are associated with high short-term mortality. The Prognostic Nutritional Index (PNI) is a simple marker reflecting both nutritional and immune status, but its prognostic value in super-elderly hip fracture patients remains unclear.

**Methods:**

We retrospectively included 614 patients aged ≥80 years with traumatic hip fractures from a tertiary hospital (*n* = 457) and the MIMIC-IV database (*n* = 157). PNI was calculated from serum albumin and absolute lymphocyte count measured within 24 h of admission. The optimal PNI cut-off for predicting 90-day all-cause mortality was determined using X-tile and used to define low- and high-PNI groups. Least absolute shrinkage and selection operator (LASSO) regression, Cox proportional hazards models, and propensity score matching (PSM) were applied to evaluate the association between admission PNI and 90-day mortality.

**Results:**

The optimal PNI cut-off was 37.2, yielding 231 patients (37.6%) in the low-PNI group (PNI ≤ 37.2) and 383 (62.4%) in the high-PNI group (PNI > 37.2). Before PSM, patients with low PNI were older and had worse laboratory profiles, including lower hemoglobin, albumin, and lymphocyte counts. The 90-day mortality rate was significantly higher in the low-PNI than in the high-PNI group (21.65% vs. 9.40%, *p* < 0.001). LASSO identified sex, race, chronic pulmonary disease, hemoglobin, creatinine, and PNI as variables associated with 90-day mortality. After 1:2 PSM, 398 patients were retained with most baseline imbalances effectively reduced. In robust Cox proportional hazards analyses for the matched cohort, high PNI was associated with lower 90-day mortality in univariate models (HR 0.34, 95% CI 0.18–0.63; *p* < 0.001); Race violated the proportional hazards assumption; this association remained robust in the fully adjusted robust Cox model with race treated as a stratified variable (HR 0.34, 95% CI 0.19–0.63; *p* < 0.001).

**Conclusion:**

A low admission PNI (≤37.2) is strongly and independently associated with higher 90-day mortality in super-elderly patients with hip fractures. PNI, derived from routine laboratory tests within 24 h of admission, provides a simple and inexpensive tool for early risk stratification in this vulnerable population.

## Graphical abstract

**Figure:**
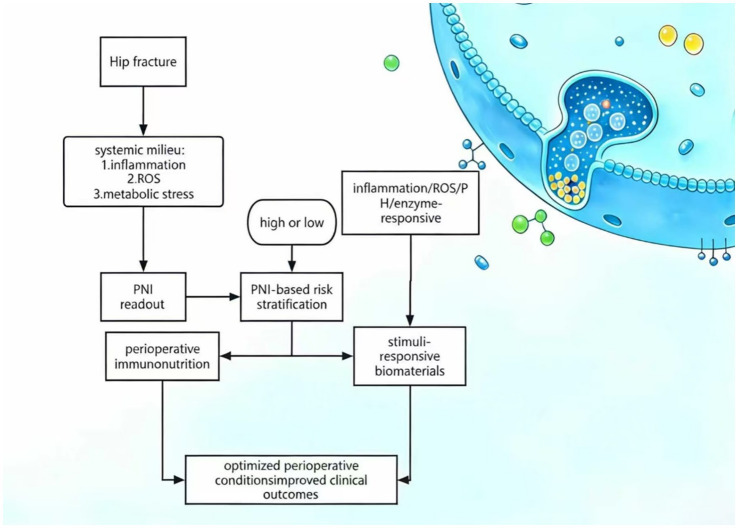

## Introduction

Super-elderly patients are uniquely vulnerable; malnutrition and sarcopenia accelerate complications (delirium, infection, mechanical ventilation) (1, 2). Hip fractures in the elderly population (years ≥ 80 years, In United Nations publications, and from definitions by the American Geriatric Society and the World Health Organization, it generally refers to people ages 80 or older) represent a significant economic and global healthcare burden, with disproportionately high rates of postoperative complications, prolonged hospitalizations, and mortality. And the mortality rates ranging from 15 to 36% within the first-year post-fracture (3–5).

Malnutrition and compromised immune function are highly prevalent in this vulnerable cohort and have been associated with increased susceptibility to infections, impaired wound healing, and poor functional outcomes (6, 7). Early identification of patients at high risk for adverse outcomes is critical for optimizing perioperative management and resource allocation, including the need for higher levels of monitoring and postoperative care.

Traditional risk stratification tools, such as the Nottingham Hip Fracture Score (NHFS) (8) and the American Society of Anesthesiologists (ASA) classification, predominantly focus on demographic and chronic comorbidity factors but often overlook the acute physiological and nutritional statuses crucial for predicting short-term prognosis in the super-elderly. The Prognostic Nutritional Index (PNI), calculated based on serum albumin concentration and peripheral lymphocyte count, has emerged as an objective, rapidly obtainable marker that reflects both the nutritional and immune status of patients (9). It is used for predicting cancer patients (10–12). Several studies have demonstrated the prognostic value of PNI in various surgical and geriatric populations; and recent evidence has highlighted the PNI, as a powerful predictor of outcomes in this vulnerable population (13).

However, data specifically targeting its early assessment in super-elderly hip fracture patients and its association with short-term outcomes, particularly mortality, in super-elderly hip fracture patients remains limited.

In this retrospective cohort study integrating electronic medical records and ICU database data from our center, we aimed to evaluate whether early measurement of PNI upon admission predicts 90-day all-cause mortality in patients aged ≥ 80 years undergoing hip fracture surgery. Given that hip fracture triggers marked systemic inflammatory and metabolic stress, PNI can be interpreted as a pragmatic, system-level readout of the admission nutrition–immune milieu. We further sought to determine a clinically actionable PNI threshold and assess the incremental value of PNI over conventional risk assessment tools to inform early triage decisions and targeted nutritional interventions, and to provide a feasible stratification variable for future studies of microenvironment-/stimuli-responsive biomaterial-enabled bone repair strategies that depend on host inflammatory and redox cues (e.g., inflammation/ROS/pH/enzyme-responsive systems).

## Methods

### Data source

This study included a total of 614 super-elderly patients with hip fractures. Among them, 457 consecutive cases were derived from the Department of Orthopedics of the Third Hospital of Guangzhou Medical University between September 1, 2017 and January 31, 2024, and 157 cases were identified from the Medical Information Mart for Intensive Care IV (MIMIC-IV, version 3.0) database. All patients from our hospital provided written informed consent prior to data collection.

The MIMIC-IV database contains comprehensive, de-identified clinical data from patients admitted to the intensive care units (ICUs) of Beth Israel Deaconess Medical Center between 2008 and 2022. Because the data are fully de-identified, the requirement for individual informed consent was waived for the MIMIC-IV cohort. One of the authors (Yuan Ye) completed the required online training and signed the data use agreement, thereby obtaining access to MIMIC-IV (Certification Number: 13149837). Variables with missing data exceeding 20% were excluded from the analysis, in accordance with the recommendations of the Strengthening Reporting of Observational Studies in Epidemiology (STROBE) statement.

### Study population

**Figure 1:**
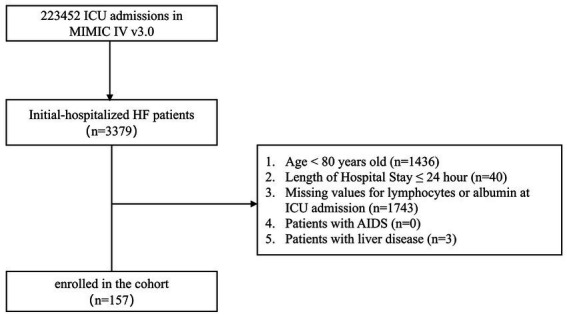
Flow chart of patient selection from the MIMIC-IV database. A total of 223,452 ICU admissions recorded in MIMIC-IV v3.0 were screened. After excluding patients aged <80 years, those with a hospital length of stay ≤24 h, those with missing lymphocyte or albumin values at ICU admission, patients with AIDS, and those with liver disease, 157 initially hospitalized hip-fracture (HF) patients were finally enrolled in the cohort.

Patients were eligible if they were aged ≥80 years and admitted with a primary diagnosis of traumatic hip fracture, identified using the International Classification of Diseases, Ninth and Tenth Revision codes (ICD-9 code 820 and ICD-10 code S72.10). Patients with pathologic fractures were excluded. We further excluded individuals without available measurements of serum albumin or absolute lymphocyte count within 24 h after admission, because these variables were required to calculate the Prognostic Nutritional Index (PNI). For patients with multiple admissions during the study period, only data from the first hospitalization were included. The patient selection process is shown in Figure 1.

### Data extraction

For the MIMIC-IV cohort, data were extracted using R statistical software (version 4.3.3; R Foundation for Statistical Computing, Vienna, Austria) in combination with Navicat Premium (version 15). For the hospital cohort, data were retrieved from the electronic medical record system using standardized query forms.

Because PNI was the primary exposure of interest, we used the first complete blood count and serum biochemistry results obtained after hospital admission (and within 24 h) to minimize the influence of subsequent treatments on laboratory values.

The following variables were collected and harmonized across the two cohorts:

- Demographics: age, sex, and race/ethnicity.
- Fracture-related variables: fracture type.
- Comorbidities: congestive heart failure (CHF), cerebrovascular disease (CBD), chronic pulmonary disease, diabetes, renal disease, cancer, Dementia.
- Laboratory parameters: white blood cell (WBC) count, absolute lymphocyte count, neutrophil count, hemoglobin, platelet count, serum creatinine, serum alanine aminotransferase (ALT), aspartate aminotransferase (AST), and serum albumin.
- Outcomes and hospital course: length of hospital stay (LOS), all-cause 90-day mortality.

The PNI was calculated according to the following formula:


$$\mathrm{PNI}=\text{serum albumin}\;(g/L)+5\times \text{absolute lymphocyte count}\;(\times 10^{9}/L)$$

### Outcome

The primary outcome of this study was all-cause mortality within 90 days of hospital admission. Vital status and date of death were obtained from the hospital information system and the MIMIC-IV follow-up records.

### Statistical analysis

In this study, the total study cohort served as the exploratory analysis set. We used it to examine baseline differences between the low and high PNI groups and the association between PNI and 90-day mortality. The matched cohort after PSM was defined as the primary analysis set, because significant imbalance in baseline characteristics existed between the two groups in the total cohort, which could introduce confounding bias. The matched cohort effectively balanced confounding factors between groups. Such confounder control at the cohort level allowed a more objective and reliable investigation of the true association between admission PNI and 90-day all-cause mortality in patients aged ≥80 years with hip fracture.

Baseline characteristics were summarized for the overall cohort. The distribution of continuous variables was assessed using the Shapiro–Wilk test. Normally distributed variables were expressed as mean ± standard deviation (SD), whereas non-normally distributed variables were presented as median and interquartile range (IQR). Between-group comparisons were performed using the Student’s *t* test or Mann–Whitney U test, as appropriate. Categorical variables were reported as counts and percentages and compared using Pearson’s chi-square test or Fisher’s exact test.

Covariates were selected based on clinical relevance and a change of at least 10% in the valid estimate. In addition, the Variance Inflation Factor (VIF) was used to assess the assumption of multicollinearity. A VIF value greater than 5 indicated the presence of multicollinearity.

Missing data was handled with the non-parametric single imputation method of random forest using the missForest package in R. All collected variables were included in the model to reduce bias. The number of decision trees was set to 100 and the random seed was set to 123 to ensure reproducibility.

The optimal cut-off value of PNI for predicting 90-day mortality was determined using X-tile software (version 3.6.1; Yale University School of Medicine). Based on this cut-off, patients were divided into a low-PNI group (PNI ≤ 37.2) and a high-PNI group (PNI > 37.2). We identified the optimal cutoff value of PNI separately in patients from the Third Affiliated Hospital of Guangzhou Medical University, and performed sensitivity analysis to test the robustness of this cutoff for outcome prediction.

To identify variables associated with 90-day mortality, we first applied least absolute shrinkage and selection operator (LASSO) regression with 10-fold cross-validation. The dataset was randomly split into a training set and a validation set in a 7:3 ratio. Variables selected by LASSO were then entered into Cox proportional hazards models to examine the association between PNI and 90-day mortality.

To further reduce baseline imbalances between the low- and high-PNI groups, we performed PSM. Variables in Table 1 were included in the propensity score model. Albumin and lymphocyte count were not used for matching because they are direct components of PNI. A 1:2 nearest-neighbor matching algorithm with a caliper width of 0.2 of the SD of the logit of the propensity score was applied. Covariate balance before and after matching was evaluated using SMDs, with SMD < 0.10 indicating adequate balance.

**Table 1:** Baseline characteristics and clinical outcomes of the overall cohort and by PNI group.

Variables	Total (*n* = 614)	Low PNI (*n* = 231)	High PNI (*n* = 383)	Statistic	*p*
Demographics					
Age, M (Q₁, Q₃)	87.00 (84.00, 90.00)	87.00 (85.00, 91.00)	86.00 (83.00, 90.00)	*Z* = −3.46	<0.001
Gender, *n* (%)				*χ*^2^ = 3.23	0.072
Male	171 (27.85)	74 (32.03)	97 (25.33)		
Female	443 (72.15)	157 (67.97)	286 (74.67)		
Race, *n* (%)				*χ*^2^ = 0.08	0.776
White	134 (21.82)	49 (21.21)	85 (22.19)		
Others	480 (78.18)	182 (78.79)	298 (77.81)		
Fracture-related variables					
Fracture types, *n* (%)				*χ*^2^ = 2.13	0.144
Femoral neck	238 (38.76)	81 (35.06)	157 (40.99)		
Intertrochanteric femoral	376 (61.24)	150 (64.94)	226 (59.01)		
Comorbidities					
CHF, *n* (%)				*χ*^2^ = 4.31	0.038
No	528 (85.99)	190 (82.25)	338 (88.25)		
Yes	86 (14.01)	41 (17.75)	45 (11.75)		
CBD, *n* (%)				*χ*^2^ = 1.04	0.308
No	527 (85.83)	194 (83.98)	333 (86.95)		
Yes	87 (14.17)	37 (16.02)	50 (13.05)		
Chronic pulmonary disease, *n* (%)				*χ*^2^ = 5.19	0.023
No	530 (86.32)	190 (82.25)	340 (88.77)		
Yes	84 (13.68)	41 (17.75)	43 (11.23)		
Diabetes, *n* (%)				*χ*^2^ = 1.39	0.239
No	465 (75.73)	181 (78.35)	284 (74.15)		
Yes	149 (24.27)	50 (21.65)	99 (25.85)		
Renal disease, *n* (%)				*χ*^2^ = 1.06	0.303
No	517 (84.20)	190 (82.25)	327 (85.38)		
Yes	97 (15.80)	41 (17.75)	56 (14.62)		
Cancer, *n* (%)				*χ*^2^ = 5.27	0.022
No	558 (90.88)	202 (87.45)	356 (92.95)		
Yes	56 (9.12)	29 (12.55)	27 (7.05)		
Dementia, *n* (%)				*χ*^2^ = 0.02	0.880
No	491 (79.97)	184 (79.65)	307 (80.16)		
Yes	123 (20.03)	47 (20.35)	76 (19.84)		
Laboratory parameters					
WBC, M (Q₁, Q₃)	9.43 (7.38, 12.09)	8.28 (6.90, 11.35)	9.85 (8.00, 12.39)	*Z* = −4.73	<0.001
Lymphocytes, M (Q₁, Q₃)	1.04 (0.75, 1.45)	0.84 (0.63, 1.12)	1.20 (0.87, 1.61)	*Z* = −9.10	<0.001
Neutrophils, M (Q₁, Q₃)	7.51 (5.62, 10.04)	6.75 (5.12, 9.75)	7.76 (6.09, 10.32)	*Z* = −3.13	0.002
Hemoglobin, Mean ± SD	105.99 ± 19.33	97.90 ± 18.37	110.87 ± 18.24	t = −8.51	<0.001
Platelets, M (Q₁, Q₃)	197.00 (156.00, 246.75)	193.00 (146.50, 254.50)	198.00 (160.00, 242.50)	*Z* = −0.59	0.556
Creatinine, M (Q₁, Q₃)	88.40 (68.00, 114.92)	89.00 (66.00, 123.76)	87.00 (68.00, 110.00)	*Z* = −0.80	0.426
ALT, M (Q₁, Q₃)	12.95 (9.53, 19.17)	12.30 (9.00, 20.05)	13.20 (10.00, 18.83)	*Z* = −0.73	0.466
AST, M (Q₁, Q₃)	20.25 (16.30, 28.00)	20.50 (16.10, 30.85)	20.10 (16.40, 26.65)	*Z* = −0.45	0.649
Albumin, M (Q₁, Q₃)	33.70 (30.00, 37.27)	29.00 (26.20, 31.00)	36.40 (33.90, 39.00)	*Z* = −18.58	<0.001
PNI, M (Q₁, Q₃)	38.98 (34.85, 43.50)	33.85 (30.85, 35.65)	42.60 (39.50, 45.70)	*Z* = −20.77	<0.001
Outcomes					
LOS, M (Q₁, Q₃)	13.00 (9.00, 17.00)	13.00 (9.00, 17.00)	13.00 (9.00, 17.00)	*Z* = −0.60	0.548
90-day all-cause mortality, *n* (%)				*χ*^2^ = 17.94	<0.001
No	528 (85.99)	181 (78.35)	347 (90.60)		
Yes	86 (14.01)	50 (21.65)	36 (9.40)		

CHF, congestive heart failure; CBD, cerebrovascular disease; WBC, white blood cell; ALT, alanine aminotransferase; AST, aspartate aminotransferase; PNI, Prognostic Nutritional Index; LOS, length of hospital stay.

Statistical tests: *t* values are from independent-samples *t* tests; *Z* values are from Mann–Whitney U tests; *χ*^2^ values are from chi-square tests.

M: median; SD: standard deviation; Q₁: first quartile; Q₃: third quartile.

In the matched cohort, robust Cox proportional hazards models were fitted to account for the paired nature of the data, and hazard ratios (HRs) with 95% confidence intervals (CIs) were reported.

In the matched cohort, robust Cox proportional hazards models were used to address clustering and the paired nature of the data, and hazard ratios (HRs) and 95% confidence intervals (CIs) were reported.

The global Schoenfeld residual test was used to verify the proportional hazards assumption for the robust Cox model in the matched cohort, and a *p* value greater than 0.05 indicated that the assumption was met.

Kaplan–Meier survival curves were generated to compare cumulative 90-day mortality between PNI groups, and differences were assessed using the log-rank test. Prespecified subgroup analyses were conducted according to age, sex, race, fracture type, CHF, CBD, dementia, chronic pulmonary disease, diabetes, renal disease and cancer. Interaction terms were tested to evaluate effect modification.

All statistical tests were two-sided, and a *p* value < 0.05 was considered statistically significant. Statistical analyses were performed using R statistical software (version 4.4.3; R Foundation for Statistical Computing, Vienna, Austria) and GraphPad Prism 8 (GraphPad Software, San Diego, CA, USA).

## Results

### Baseline demographics and clinical characteristics before and after PSM

**Figure 2:**
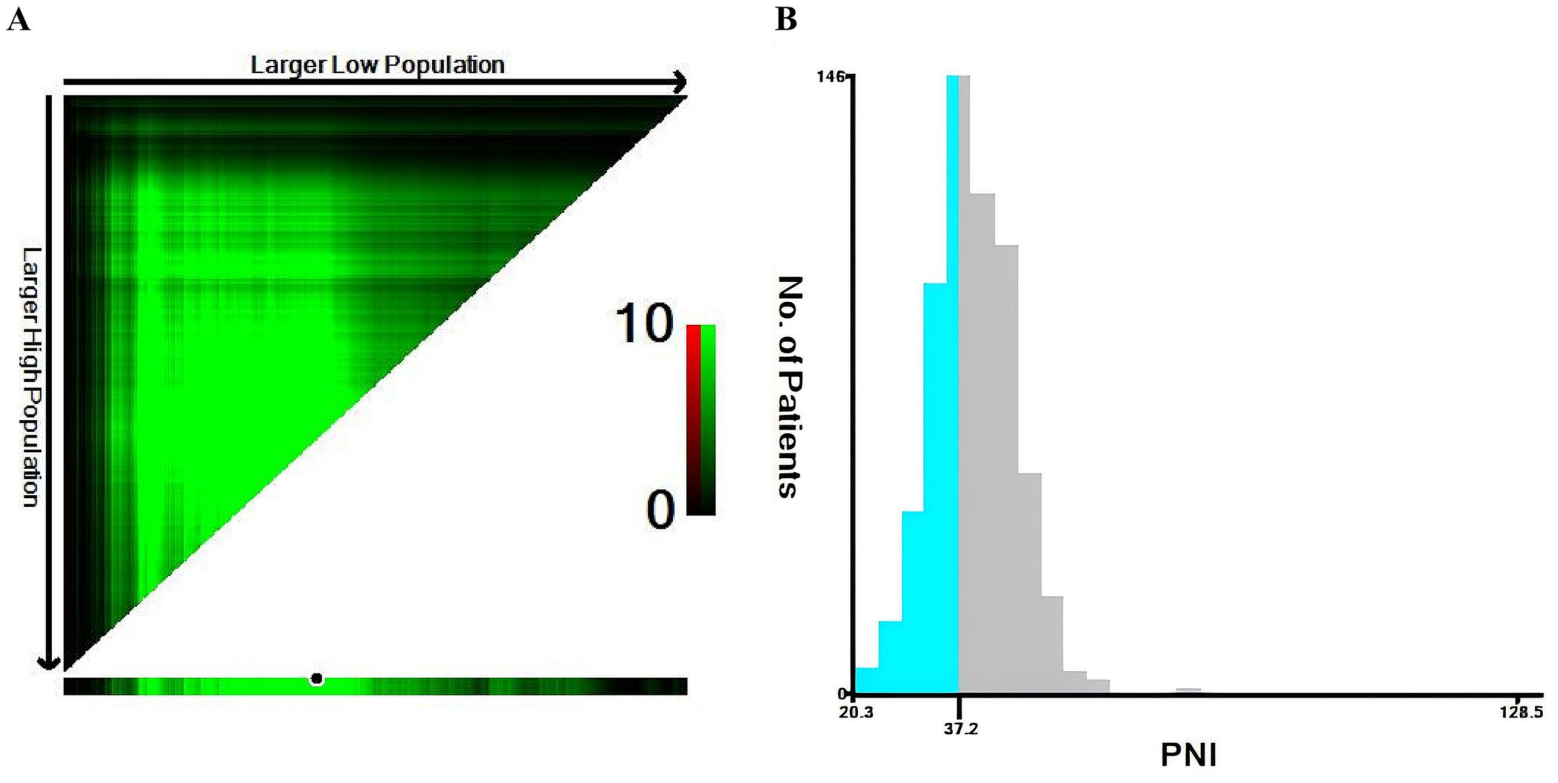
Determination of the optimal PNI cut-off value using X-tile. **(A)** X-tile plots showing the relationship between different PNI cut-off points and 90-day all-cause mortality. The color scale from green to red indicates increasing log-rank chi-square values. **(B)** Histogram of the PNI distribution with the optimal cut-off value of 37.2 (vertical line) identified by X-tile, which was used to define the low-PNI and high-PNI groups.

A total of 614 patients with hip fractures who met the inclusion and exclusion criteria were enrolled (Figure 1). The median age was 87.0 years (IQR 84.0–90.0), and 443 patients (72.15%) were female. Using X-tile analysis, a PNI value of 37.2 was identified as the optimal cut-off for predicting 90-day mortality (Figure 2). Accordingly, patients were divided into a low-PNI group (PNI ≤ 37.2, *n* = 231) and a high-PNI group (PNI > 37.2, *n* = 383). To verify the robustness of the predictive performance of the PNI cutoff value, we separately determined the optimal PNI cutoff in 457 participants from our center using the same method. The optimal PNI cutoff for predicting 90-day all-cause mortality in this local population was 37.5, which was highly similar to that of the total cohort. Six covariates had missing data in this study, including ALT, AST, Creatinine, WBC, Hemoglobin and Platelet. The missing rate was 6.84% for ALT and 14.98% for AST, and all other covariates had missing rates below 5%.

Baseline characteristics of the two groups are summarized in Table 1. Before PSM, patients in the low-PNI group were older (87.00 vs. 86.00 years, *p* < 0.001), had lower hemoglobin concentrations (97.90 ± 18.37 vs. 110.87 ± 18.24 g/L, *p* < 0.001) compared with those in the high-PNI group, and laboratory analyses also showed significant between-group differences in WBC count (8.28 vs. 9.85 × 10^9^/L, *p* < 0.001), neutrophil count (6.75 vs. 7.76 × 10^9^/L, *p* = 0.002), lymphocyte count (0.84 vs. 1.20 × 10^9^/L, *p* < 0.001), and serum albumin (29.00 vs. 36.40 g/L, *p* < 0.001); all of these parameters were lower in the low-PNI group.

The prevalence of CHF (17.75% vs. 11.15%, *p* = 0.038), Chronic Pulmonary Disease (17.75% vs. 11.23%, *p* = 0.023) and Cancer (12.55% vs. 7.05%, *p* = 0.022) was higher in the low-PNI group than in the high-PNI group. Patients with low PNI had a significantly higher 90-day all-cause mortality rate than those with high PNI (21.65% vs. 9.40%, *p* < 0.001), indicating that low PNI was associated with worse prognosis.

**Figure 3:**
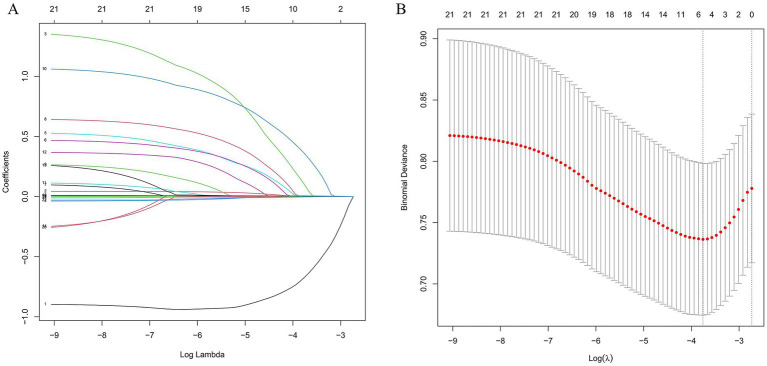
Variable selection by LASSO regression. **(A)** LASSO coefficient profiles of the candidate predictors for 90-day all-cause mortality as a function of log(*λ*). **(B)** Ten-fold cross-validation for selection of the optimal penalization parameter *λ*. The dotted vertical line indicates the value of *λ* that minimizes the cross-validated binomial deviance and corresponds to the final set of selected variables.

All baseline variables were entered into LASSO regression with 10-fold cross-validation. The optimal penalty parameter *λ* was 0.023342 (Figure 3). Six covariates were ultimately identified as being associated with 90-day mortality: gender, race, chronic pulmonary disease, hemoglobin, creatinine and PNI. Variance inflation factor values for continuous covariates were all less than 10, indicating no severe multicollinearity.

**Figure 4:**
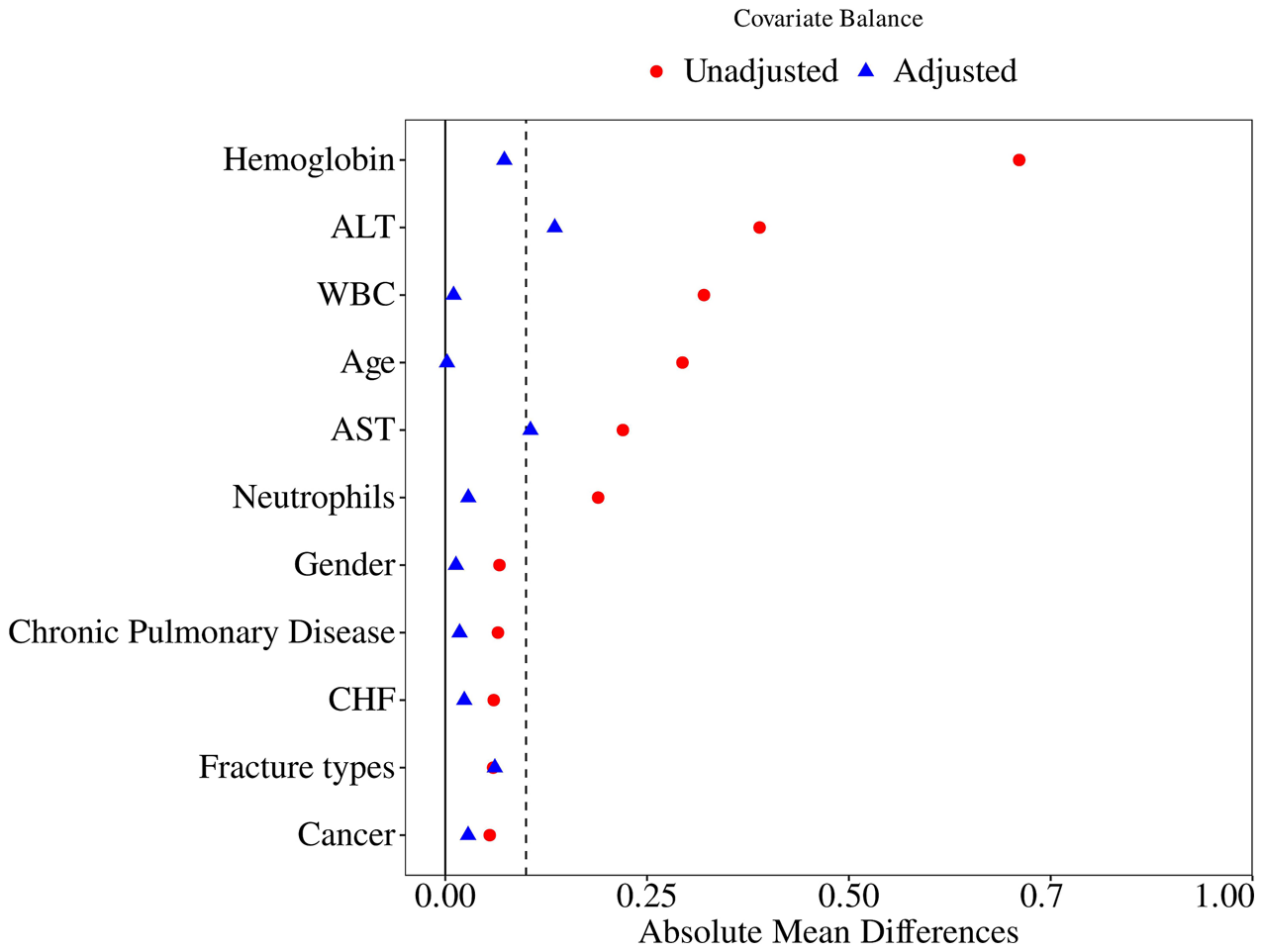
Covariate balance before and after propensity score matching. Standardized mean differences (SMDs) of key baseline covariates between low- and high-PNI groups before (red circles) and after (blue triangles) propensity score matching are displayed. The vertical dashed line at SMD = 0.10 denotes the threshold for acceptable balance. Most covariates achieved adequate balance after matching.

Variables in Table 1 were included in the propensity score model. Serum albumin and lymphocyte count—direct components of PNI—were not used for matching. A 1:2 nearest-neighbor matching algorithm with a caliper width of 0.2 was applied. After PSM, 398 patients (216 in low PNI group, 182 in high PNI group) were retained for further analysis, and most baseline imbalances were substantially reduced (Table 2). Although a 1:2 matching scheme was specified *a priori*, some low-PNI patients had fewer than two eligible matches within the caliper; thus, the final matched sample size reflects the common support and matching constraints. The SMDs before and after matching are shown in Figure 4. Even after PSM, the 90-day mortality rate remained significantly higher in the low-PNI group than in the high-PNI group (21.30% vs. 7.69%, *p* < 0.001).

**Table 2:** Comparison of baseline characteristics before and after propensity score matching according to PNI group.

Variable	Before PSM						After PSM					
	Total (*n* = 614)	Low PNI (*n* = 231)	High PNI (*n* = 383)	Statistic	*p*	SMD	Total (*n* = 398)	Low PNI (*n* = 216)	High PNI (*n* = 182)	Statistic	*p*	SMD
Demographics												
Age, M (Q₁, Q₃)	87.00 (84.00, 90.00)	87.00 (85.00, 91.00)	86.00 (83.00, 90.00)	*Z* = −3.462	<0.001	−0.294	87.00 (84.00, 91.00)	87.00 (85.00, 91.00)	87.00 (84.00, 90.00)	*Z* = −0.850	0.395	−0.064
Gender, *n* (%)				*χ*^2^ = 3.227	0.072					*χ*^2^ = 0.425	0.514	
Male	171 (27.85)	74 (32.03)	97 (25.33)			−0.154	118 (29.65)	67 (31.02)	51 (28.02)			−0.067
Female	443 (72.15)	157 (67.97)	286 (74.67)			0.154	280 (70.35)	149 (68.98)	131 (71.98)			0.067
Race, *n* (%)				*χ*^2^ = 0.081	0.776					*χ*^2^ = 0.202	0.653	
White	134 (21.82)	49 (21.21)	85 (22.19)			0.024	76 (19.1)	43 (19.91)	33 (18.13)			−0.046
Others	480 (78.18)	182 (78.79)	298 (77.81)			−0.024	322 (80.9)	173 (80.09)	149 (81.87)			0.046
Fracture-related variables												
Fracture types, *n* (%)				*χ*^2^ = 2.133	0.144					*χ*^2^ = 0.252	0.616	
Femoral neck	238 (38.76)	81 (35.06)	157 (40.99)			0.121	143 (35.93)	80 (37.04)	63 (34.62)			−0.051
Intertrochanteric femoral	376 (61.24)	150 (64.94)	226 (59.01)			−0.121	255 (64.07)	136 (62.96)	119 (65.38)			0.051
Comorbidities												
CHF, *n* (%)				*χ*^2^ = 4.306	0.038					*χ*^2^ = 1.557	0.212	
No	528 (85.99)	190 (82.25)	338 (88.25)			0.186	338 (84.92)	179 (82.87)	159 (87.36)			0.135
Yes	86 (14.01)	41 (17.75)	45 (11.75)			−0.186	60 (15.08)	37 (17.13)	23 (12.64)			−0.135
CBD, *n* (%)				*χ*^2^ = 1.040	0.308					*χ*^2^ = 3.868	0.049	
No	527 (85.83)	194 (83.98)	333 (86.95)			0.088	344 (86.43)	180 (83.33)	164 (90.11)			0.227
Yes	87 (14.17)	37 (16.02)	50 (13.05)			−0.088	54 (13.57)	36 (16.67)	18 (9.89)			−0.227
Chronic pulmonary disease, *n* (%)				*χ*^2^ = 5.190	0.023					*χ*^2^ = 0.600	0.439	
No	530 (86.32)	190 (82.25)	340 (88.77)			0.207	335 (84.17)	179 (82.87)	156 (85.71)			0.081
Yes	84 (13.68)	41 (17.75)	43 (11.23)			−0.207	63 (15.83)	37 (17.13)	26 (14.29)			−0.081
Diabetes, *n* (%)				*χ*^2^ = 1.385	0.239					*χ*^2^ = 1.105	0.293	
No	465 (75.73)	181 (78.35)	284 (74.15)			−0.096	307 (77.14)	171 (79.17)	136 (74.73)			−0.102
Yes	149 (24.27)	50 (21.65)	99 (25.85)			0.096	91 (22.86)	45 (20.83)	46 (25.27)			0.102
Renal disease, *n* (%)				*χ*^2^ = 1.060	0.303					*χ*^2^ = 0.102	0.749	
No	517 (84.2)	190 (82.25)	327 (85.38)			0.089	332 (83.42)	179 (82.87)	153 (84.07)			0.033
Yes	97 (15.8)	41 (17.75)	56 (14.62)			−0.089	66 (16.58)	37 (17.13)	29 (15.93)			−0.033
Cancer, *n* (%)				*χ*^2^ = 5.267	0.022					*χ*^2^ = 1.475	0.225	
No	558 (90.88)	202 (87.45)	356 (92.95)			0.215	362 (90.95)	193 (89.35)	169 (92.86)			0.136
Yes	56 (9.12)	29 (12.55)	27 (7.05)			−0.215	36 (9.05)	23 (10.65)	13 (7.14)			−0.136
Dementia, *n* (%)				*χ*^2^ = 0.023	0.880					*χ*^2^ = 0.719	0.396	
No	491 (79.97)	184 (79.65)	307 (80.16)			0.013	323 (81.16)	172 (79.63)	151 (82.97)			0.089
Yes	123 (20.03)	47 (20.35)	76 (19.84)			−0.013	75 (18.84)	44 (20.37)	31 (17.03)			−0.089
Laboratory parameters												
WBC, M (Q₁, Q₃)	9.43 (7.38, 12.09)	8.28 (6.90, 11.35)	9.85 (8.00, 12.39)	*Z* = −4.734	<0.001	0.320	8.81 (7.00, 11.59)	8.49 (6.91, 11.33)	9.07 (7.32, 11.78)	*Z* = −1.630	0.103	0.099
Neutrophils, M (Q₁, Q₃)	7.51 (5.62, 10.04)	6.75 (5.12, 9.75)	7.76 (6.09, 10.32)	*Z* = −3.131	0.002	0.189	6.95 (5.19, 9.83)	6.86 (5.14, 9.74)	6.98 (5.51, 10.14)	*Z* = −0.672	0.502	0.017
Hemoglobin, Mean ± SD	105.99 ± 19.33	97.90 ± 18.37	110.87 ± 18.24	t = −8.513	<0.001	0.711	100.86 ± 18.34	99.21 ± 17.90	102.82 ± 18.71	t = −1.961	0.051	0.193
Platelets, M (Q₁, Q₃)	197.00 (156.00, 246.75)	193.00 (146.50, 254.50)	198.00 (160.00, 242.50)	*Z* = −0.589	0.556	−0.075	189.00 (150.00, 248.50)	194.50 (149.75, 259.50)	187.50 (150.00, 236.50)	*Z* = −0.883	0.377	−0.184
Creatinine, M (Q₁, Q₃)	88.40 (68.00, 114.92)	89.00 (66.00, 123.76)	87.00 (68.00, 110.00)	*Z* = −0.797	0.426	−0.074	91.50 (68.00, 122.00)	88.40 (65.00, 120.00)	94.50 (71.00, 123.00)	*Z* = −1.700	0.089	0.118
ALT, M (Q₁, Q₃)	12.95 (9.53, 19.17)	12.30 (9.00, 20.05)	13.20 (10.00, 18.83)	*Z* = −0.729	0.466	−0.389	12.45 (9.22, 19.00)	12.30 (9.00, 19.15)	12.75 (9.62, 18.69)	*Z* = −0.157	0.875	−0.113
AST, M (Q₁, Q₃)	20.25 (16.30, 28.00)	20.50 (16.10, 30.85)	20.10 (16.40, 26.65)	*Z* = −0.455	0.649	−0.220	20.10 (16.33, 28.48)	20.40 (16.17, 29.56)	19.75 (16.55, 27.00)	*Z* = −0.460	0.646	−0.136
Outcomes												
LOS, M (Q₁, Q₃)	13.00 (9.00, 17.00)	13.00 (9.00, 17.00)	13.00 (9.00, 17.00)	*Z* = −0.600	0.548		13.29 (9.00, 17.00)	13.03 (9.00, 17.00)	13.76 (9.32, 17.00)	*Z* = −0.38	0.706	
90-day all-cause mortality, *n* (%)				*χ*^2^ = 17.94	<0.001					*χ*^2^ = 14.28	<0.001	
No	528 (85.99)	181 (78.35)	347 (90.60)				338 (84.92)	170 (78.70)	168 (92.31)			
Yes	86 (14.01)	50 (21.65)	36 (9.40)				60 (15.08)	46 (21.30)	14 (7.69)			

PSM, propensity score matching; PNI, Prognostic Nutritional Index; CHF, congestive heart failure; CBD, cerebrovascular disease; WBC, white blood cell; ALT, alanine aminotransferase; AST, aspartate aminotransferase; LOS, length of stay; SMD, standardized mean difference.

Statistical tests: *t* values are from independent-samples *t*-tests; *Z* values are from Mann–Whitney U tests; *χ*^2^ values are from chi-square tests.

Balance criterion: SMD values < 0.10 were considered to indicate adequate covariate balance.

### Kaplan–Meier curves

**Figure 5:**
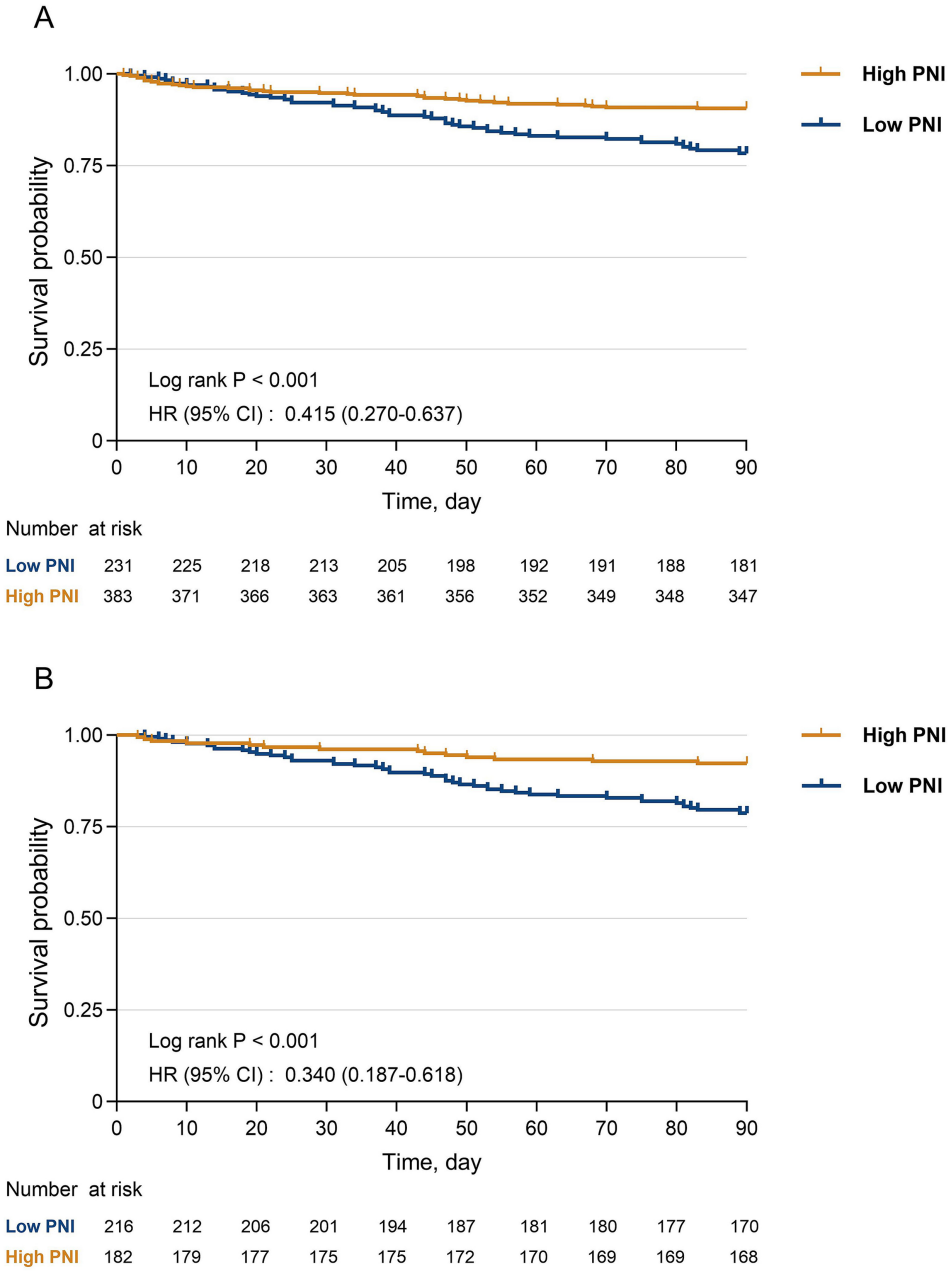
Kaplan–Meier curves for 90-day all-cause mortality according to PNI group. **(A)** Unmatched cohort (*n* = 614). **(B)** Propensity score–matched cohort (*n* = 398). Survival probability over 90 days is plotted for patients with low PNI (PNI ≤ 37.2) and high PNI (PNI > 37.2). Log-rank p values and hazard ratios (HRs) with 95% confidence intervals (CIs) from Cox models are shown in each panel. Numbers at risk at selected time points are provided below the *x*-axis.

Kaplan–Meier survival analyses demonstrated that patients with higher PNI had better 90-day survival than those with lower PNI in the overall cohort, although the survival curves crossed during the early follow-up period (approximately days 0–20). After PSM, the separation between the survival curves became more stable, and the pattern remained consistent with that observed in the unmatched cohort (Figure 5).

### PNI as an independent risk factor for 90-day all-cause mortality

In the matched cohort, univariate robust Cox proportional hazards analysis showed that, with the low-PNI group as the reference, higher PNI was significantly associated with a reduced risk of 90-day all-cause mortality (HR 0.34, 95% CI 0.18–0.63; *p* < 0.001). In multivariable robust Cox models, higher PNI (HR 0.33, 95% CI 0.17–0.64; *p* < 0.001) remained an independent predictor of 90-day all-cause mortality (Table 3).

**Table 3:** Univariate and multivariable robust Cox proportional hazards regression analysis for 90-day all-cause mortality after propensity score matching.

Outcomes	Univariate analysis					Multivariate analysis				
	*β*	S.E	*Z*	*p*	HR (95%CI)	*β*	S.E	*Z*	*p*	HR (95%CI)
90-day mortality										
Low PNI					1.00 (Reference)					1.00 (Reference)
High PNI	−1.08	0.31	−3.46	**<0.001**	0.34 (0.18–0.63)	−1.10	0.33	−3.31	<0.001	0.33 (0.17–0.64)

PNI, Prognostic Nutritional Index; HR, hazard ratio; CI, confidence interval; SE, standard error.

Robust Cox proportional hazards models were fitted in the propensity score–matched cohort (*n* = 398). The low-PNI group (PNI ≤ 37.2) served as the reference category.

Multivariable Cox models were then built stepwise. In the crude model (Model 1), high PNI was associated with a 66% lower hazard of 90-day death (HR 0.34, 95% CI 0.19–0.62; *p* < 0.001). After adjustment for sex, race, and age (Model 2), high PNI remained significantly associated with reduced 90-day mortality (HR 0.35, 95% CI 0.19–0.63; *p* < 0.001). In the fully adjusted model, Following adjustment for covariates selected by Lasso regression and clinically relevant covariates (Model 3), high PNI continued to be independently associated with a lower risk of 90-day mortality (HR 0.34, 95% CI 0.18–0.62; *p* < 0.001) (Table 4).

**Table 4:** Cox regression models for the association between PNI and 90-day all-cause mortality after propensity score matching.

Outcomes	Model 1		Model 2		Model 3		Model 4	
	HR (95%CI)	*p*	HR (95%CI)	*p*	HR (95%CI)	*p*	HR (95%CI)	*p*
90-day mortality								
Low PNI	1.00 (Reference)		1.00 (Reference)		1.00 (Reference)		1.00 (Reference)	
High PNI	0.34 (0.19–0.62)	<0.001	0.35 (0.19–0.63)	<0.001	0.32 (0.17–0.58)	<0.001	0.31 (0.17–0.58)	<0.001

PNI, Prognostic Nutritional Index; HR, hazard ratio; CI, confidence interval.

Model 1: Crude model including PNI only.

Model 2: Adjusted for sex, race, and age.

Model 3: Additionally adjusted for sex, race, fracture types, chronic pulmonary disease, age, WBC, hemoglobin and creatinine.

Model 4: Analyses stratified by two racial groups and independently fitted based on Model 3.

We used the global Schoenfeld residual test to assess the proportional hazards assumption for the final Cox model (Model 3). Race was the only variable that violated the assumption. In the fully adjusted model (Model 4), race was treated as a stratified variable, and the global Schoenfeld residual test yielded a *p* value of 0.49, indicating that the model satisfied the proportional hazards assumption. Detailed results for both Schoenfeld tests are presented in Supplementary Table 1. In Model 4, high PNI remained an independent protective factor against 90-day mortality (HR 0.34, 95% CI 0.19–0.63, *p* < 0.001; Table 4).

### Subgroup analyses

**Figure 6:**
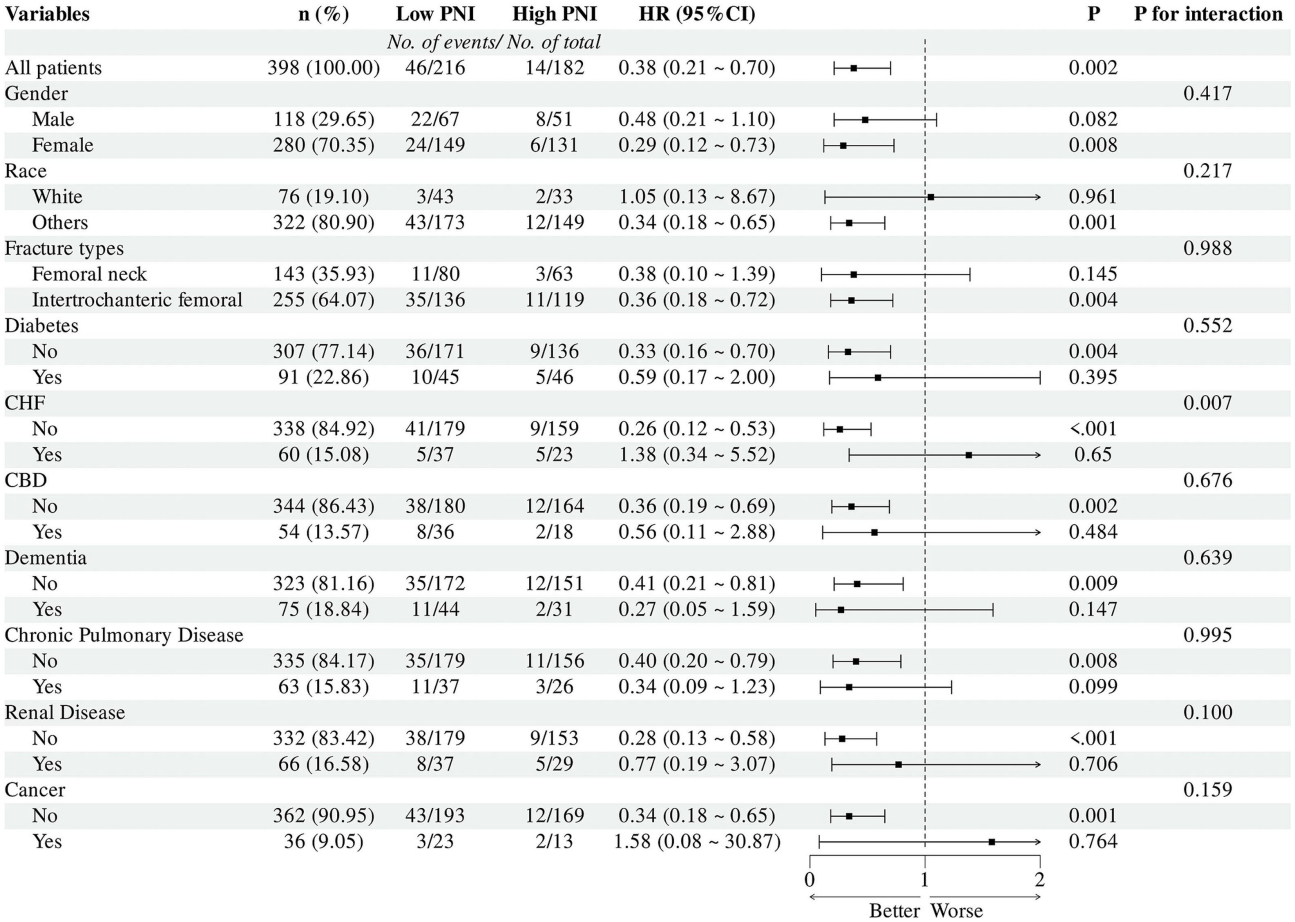
Subgroup analyses of the association between PNI and 90-day all-cause mortality. Forest plot of hazard ratios (HRs) and 95% confidence intervals (CIs) for high versus low PNI in prespecified subgroups within the propensity score–matched cohort (*n* = 398). The number of events and total number of patients in each PNI group are shown for each subgroup. The vertical dashed line represents the overall HR. The rightmost column reports *p* values for interaction between PNI category and each subgroup variable.

Subgroup analyses were conducted to evaluate the consistency of the association between PNI and 90-day mortality across strata defined by age, sex, race, fracture type, CHF, cerebrovascular disease, dementia, chronic pulmonary disease, diabetes, renal disease, and cancer (Figure 6). Forest plots showed that this association was generally consistent across most subgroups, with no statistically significant interactions detected between PNI and the majority of subgroup variables (all interaction *p* > 0.05). A statistically significant interaction was observed for CHF (interaction *p* = 0.007). Higher PNI had a more pronounced protective effect in patients without CHF. These findings support the robustness of the association between PNI and 90-day mortality in super-elderly patients with hip fractures.

## Discussion

Our findings indicate that, among super-elderly patients with hip fractures, a low admission Prognostic Nutritional Index (PNI ≤ 37.2) is closely associated with increased 90-day all-cause mortality. This association remained robust after PSM and multivariable adjustment for demographic characteristics, comorbidities, and laboratory parameters. In this bicenter cohort of 614 eligible patients, X-tile analysis identified 37.2 as the optimal PNI cut-off for predicting 90-day mortality. When patients were stratified into low- and high-PNI groups according to this threshold, the 90-day mortality rate was consistently higher in the low-PNI group than in the high-PNI group, both before and after PSM. Robust Cox proportional hazards regression further demonstrated that higher PNI is an independent protective factor for 90-day survival, and this protective effect persisted even after full adjustment for potential confounders. Subgroup analyses showed that the association between PNI and mortality was generally consistent across most strata, except among patients with congestive heart failure (CHF), underscoring the stability of PNI as a prognostic marker. These findings indicate that early PNI reflects a dimension of risk that is not fully captured by traditional clinical factors in super-elderly hip-fracture patients (14, 15). These patterns support the notion that low PNI represents a state of combined malnutrition and immune dysfunction rather than simply reflecting differences in fracture pattern or comorbidity burden (16).

Compared with younger patients with hip fractures, super-elderly individuals have substantially higher risks of pneumonia, urinary tract infection, acute kidney injury, subsequent cognitive impairment, and in-hospital mortality (17, 18). They are also more likely to experience loss of independence and multiple complications. A multicenter study reported that only 63.4% of super-elderly patients regained their pre-fracture level of independence, whereas the 3-month mortality rate reached 33% (19). Accurate risk stratification is therefore crucial to optimize management in this highly vulnerable population. Previous work has shown that a comprehensive care model including nutritional support, infection prevention, pressure-ulcer and thrombosis prophylaxis, and individualized management of comorbidities can significantly reduce postoperative mortality and related complications in super-elderly hip-fracture patients (20).

Approximately 70% of older adults with hip fractures have underlying respiratory, neurovascular, psychological, cardiovascular, or endocrine disorders. The complexity of perioperative management lies in coordinating care for multiple coexisting conditions, which is also a key determinant of surgical decision-making and postoperative recovery (15). However, conventional prognostic scores have important limitations and may not meet the assessment needs of this highly frail group. The Nottingham Hip Fracture Score (NHFS), a hip-fracture–specific tool, was developed primarily to predict 30-day mortality (21, 22) and was derived from cohorts of patients mostly younger than 65 years (23). Its variables focus on age, number of comorbidities, and fracture type, but do not capture core internal milieu parameters such as nutritional reserve and immune function, which are fundamental for super-elderly patients to tolerate trauma, surgery, and complications. As a result, its ability to identify truly high-risk individuals is limited. The American Society of Anesthesiologists (ASA) physical status classification is centered on perioperative anesthetic risk, emphasizing overall organ function and surgical tolerance, but it provides little information on long-term functional recovery or nutrition-related complications (e.g., infection, pressure ulcers) and is subject to inter-observer variability.

A systematic review comparing various prediction tools in hip-fracture populations (24) found that the Frail-VIG index had the highest ability to predict 1-year mortality (AUC 0.91). The Frail-VIG index incorporates 22 variables from five domains (clinical, nutritional, functional, cognitive, and social), and the inclusion of nutrition-related items appears to be a key advantage over other scores. These findings highlight the importance of nutritional status for long-term prognosis in hip-fracture patients. Other studies have also shown that both short-term and long-term mortality after hip fracture are significantly associated with poor nutritional status at admission (25, 26).

PNI is a composite index integrating serum albumin and lymphocyte count that has demonstrated good prognostic performance in oncology, critical care, and orthopedics (27–29). Its major strength lies in capturing two fundamental physiological dimensions-nutritional reserve and immune competence—rather than a single clinical characteristic. Unlike traditional scores that focus mainly on overt clinical factors, PNI reflects the underlying nutrition-immune status that enables super-elderly patients to maintain organ function and tolerate trauma and infection. This may explain why PNI provides prognostic information beyond conventional risk factors. Previous studies have reported that low PNI is significantly associated with in-hospital, 30-day, and 1-year mortality in patients with femoral fractures and may outperform other composite indices such as the HALP score and the systemic immune-inflammation index (SII) (30). A meta-analysis of eight studies including 11,576 older adults showed that patients with higher PNI had significantly lower mortality than those with lower PNI (31). Consistent with these reports, our results also demonstrate that patients with low PNI have substantially higher short-term mortality than those with high PNI.

The association between low PNI and adverse outcomes can be explained by plausible biological mechanisms. Serum albumin is a key marker of visceral protein stores; decreased albumin reflects protein–energy malnutrition and/or systemic inflammation. Hypoalbuminemia directly contributes to delayed wound healing (32), loss of muscle mass (33), impaired immune function (34), and reduced cardiopulmonary reserve (35), which in turn delay mobilization and increase the risk of complications such as infection, pressure ulcers, and venous thromboembolism (36, 37). Lymphocyte count is an important indicator of cell-mediated immunity; lymphopenia suggests immunosuppression and heightened stress responses, making patients more susceptible to infections and sepsis after the combined insult of fracture and surgery, and impairing their ability to maintain organ perfusion and functional recovery (38). Importantly, in our multivariable models adjusting for comorbidities and laboratory indices, PNI remained an independent predictor of mortality, suggesting that it captures a dimension of frailty not fully represented by traditional risk scores and reinforcing its incremental prognostic value.

In our study, the Kaplan–Meier survival curves showed a noteworthy pattern. In the unmatched cohort, the survival trajectories of the low- and high-PNI groups crossed during the early follow-up period (approximately the first 20 days), whereas after PSM this crossing was attenuated and the separation between curves became more stable. Previous studies have reported that death after hip fracture is mainly attributable to cardiovascular events and pneumonia, and that postoperative complications such as delirium, pneumonia, acute cardiovascular events, and pulmonary embolism are important contributing factors (39). Compared with younger patients, super-elderly individuals have higher prevalences of hypertension, dementia, and ischemic heart disease, receive transfusions more frequently (17), and more often present with ASA class 4–5 and Elixhauser comorbidity scores ≥6, indicating poorer baseline organ reserve, lower tolerance to surgical and anesthetic stress, and a greater likelihood of early acute complications (40). These acute events are only weakly influenced by baseline nutritional status, whereas longer-term prognosis depends more on recovery capacity supported by adequate nutrition and immune function. This helps to explain why the survival difference between the two PNI groups widens over time. The reduction in baseline covariate imbalance after PSM further supports the notion that initial differences in comorbidities and treatment strategies partly account for the early crossing of the survival curves, whereas the nutrition-immune status reflected by PNI is a key determinant of longer-term outcomes.

Subgroup analyses further enhanced the clinical applicability of PNI. Its association with 90-day mortality remained consistent across most strata, including age, sex, fracture type, diabetes, and cerebrovascular disease, confirming the broad predictive utility of PNI. The only significant interaction was observed in the CHF subgroup, where the protective effect of high PNI was more pronounced in patients without CHF. This difference may be explained by the pathophysiological characteristics of CHF: volume overload can lead to hemodilution and altered vascular permeability, thereby lowering serum albumin concentrations (41). Cardiac dysfunction also causes systemic venous congestion, including impaired hepatic venous outflow, ultimately resulting in hepatic congestion and heart failure–related hepatic abnormalities. Sinusoidal dilatation compresses hepatocytes and induces hepatocellular atrophy and mitochondrial dysfunction, which reduce adenosine triphosphate (ATP) synthesis and suppress albumin production (42), making PNI a less accurate reflection of true nutritional reserves in patients with CHF. By contrast, in patients without CHF, PNI more faithfully represents nutrition-immune function and thus has greater predictive value.

Several limitations of this study should be acknowledged. First, the key components of PNI—serum albumin and lymphocyte count—are susceptible to the influence of fracture- and surgery-related trauma stress and acute inflammatory responses, which may transiently deviate from baseline and introduce misclassification in early risk stratification. However, this sensitivity also supports interpreting PNI as a pragmatic, system-level readout of the admission nutrition–immune milieu, which is the context in which perioperative, microenvironment-guided strategies (including stimuli-responsive biomaterial approaches) would be implemented. Second, PNI is a static baseline measure and cannot capture dynamic changes related to nutritional support, complication control, or rehabilitation during hospitalization, thereby limiting its ability to reflect the full trajectory of prognosis. Future studies should assess serial PNI measurements and evaluate whether changes in PNI track clinical course and recovery. Third, this was a bicenter cohort study, and selection bias cannot be completely excluded. We did not collect detailed information on dietary intake, pre-fracture functional status, or social support, which may affect both PNI and outcomes and thus restrict the generalizability of our findings to other settings. Finally, current evidence, including our study, is observational and demonstrates only an association between PNI and prognosis; prospective interventional trials are needed to determine whether improving PNI can directly reduce mortality and whether PNI can serve as a stratification factor or effect modifier in trials of microenvironment-/stimuli-responsive regenerative interventions.

Surgery-related variables (timing, procedure type, and anesthesia) were not included by design because our primary objective was to evaluate the prognostic value of admission PNI (within 24 h) for early risk stratification before downstream in-hospital interventions are determined. After rigorous adjustment for baseline confounders using 10-fold cross-validated LASSO selection and additional PSM balancing, PNI remained independently associated with 90-day mortality in robust multivariable Cox models (HR = 0.34, 95% CI 0.19–0.63; *p* < 0.001), supporting the robustness of our main conclusion despite the absence of surgery-related covariates. Nevertheless, future prospective studies should collect and model surgical timing and approach to further delineate their interplay with baseline immunonutritional status.

Despite these limitations, our study has important clinical implications. PNI is calculated from routine, inexpensive, and widely available laboratory tests and can be easily implemented in daily practice. The identification of a clear optimal cut-off value (37.2) facilitates rapid clinical application. Incorporating PNI into early risk stratification for patients aged ≥80 years with hip-fracture may help clinicians promptly identify high-risk individuals and formulate individualized strategies, including intensified monitoring, optimization of hemodynamics and comorbidities, and early targeted nutritional and rehabilitation interventions. Beyond prognostication, PNI may serve as a clinically accessible, system-level “environment-responsive” indicator of the peri-fracture nutrition–immune milieu, which is relevant for translational efforts in microenvironment-/stimuli-responsive biomaterials for bone and cartilage repair.

Although causal inferences cannot be drawn from our observational design, the consistent association between higher PNI and lower mortality across multiple adjusted models and subgroups provides a strong rationale for future prospective trials evaluating structured perioperative nutritional support and immunonutrition, particularly in patients with low baseline PNI. Importantly, future biomaterial-focused studies could incorporate PNI as a pre-specified stratification factor and evaluate treatment-by-milieu interactions for interventions such as inflammation/ROS-responsive scaffolds or hydrogels combined with perioperative immunonutrition. In summary, low admission PNI in patients aged ≥80 years with hip fracture reflects marked disturbances in nutritional and immune status and is independently associated with increased 90-day all-cause mortality, independent of age, comorbidities, and conventional laboratory indices. Early assessment of PNI offers a simple and practical tool for risk stratification and may help optimize individualized perioperative management and improve short-term outcomes in this rapidly growing, highly frail population.

Implications for microenvironment-/stimuli-responsive biomaterials and trial design.

From a translational perspective, admission PNI may be viewed as a pragmatic, system-level readout of the peri-fracture nutrition–immune milieu, which could influence host inflammatory and redox cues relevant to microenvironment-/stimuli-responsive biomaterials. Accordingly, PNI may be incorporated into future biomaterial-oriented studies in at least three ways: (1) as a pre-specified stratification factor for enrollment and/or randomization (e.g., PNI ≤ 37.2 vs. >37.2); (2) to test treatment-by-milieu interactions (treatment × PNI) to evaluate effect modification; and (3) as a baseline covariate for risk adjustment to improve interpretability and comparability across cohorts.

## Data Availability

The datasets used and/or analyzed during the current study are available from the corresponding author on reasonable request. And the data used to support the findings of this study can be obtained from the MIMIC-IV database (https://mimic.physionet.org/).
