# Supplementary material for: Prognostic nutritional index at admission predicts 90-day mortality in patients aged ≥80 years with hip fracture: a system-level readout of the nutrition–immune milieu relevant to microenvironment-responsive bone repair
Source: Front Med (Lausanne). 2026 Apr 17;13:1788594. doi: 10.3389/fmed.2026.1788594 (PMC13132761; doi:10.3389/fmed.2026.1788594)
Supplement: Supplementary file 1 [file Table_1.docx]

|  |
| --- |

### Table S1: Schoenfeld Residual Test for Proportional Hazards Assumption in Model 3 (Robust Cox Regression After Propensity Score Matching).

| Variable | Unstratified Model 3 | | | Stratified Model 3 (by Race) | | |
| --- | --- | --- | --- | --- | --- | --- |
|  | χ2 | df | P value | χ2 | df | P value |
| PNI group | 1.893 | 1 | 0.169 | 1.556 | 1 | 0.210 |
| Gender | 0.376 | 1 | 0.540 | 0.193 | 1 | 0.660 |
| Race | 13.257 | 1 | <0.001 | Stratification variable | - | - |
| Chronic pulmonary disease | 0.501 | 1 | 0.479 | 1.260 | 1 | 0.260 |
| Hemoglobin | 2.159 | 1 | 0.141 | 1.303 | 1 | 0.250 |
| Creatinine | 0.185 | 1 | 0.667 | 0.985 | 1 | 0.320 |
| Fracture type | 0.394 | 1 | 0.530 | 0.231 | 1 | 0.630 |
| Age | 2.001 | 1 | 0.157 | 2.405 | 1 | 0.120 |
| White blood cell count | 2.416 | 1 | 0.120 | 2.006 | 1 | 0.160 |
| **Global** | **23.837** | **9** | **0.005** | **10.137** | **8** | **0.260** |

|  |
| --- |

|  |
| --- |

Values are from Schoenfeld residual tests for the Cox proportional hazards assumption. Model 3 corresponds to the robust Cox model in the propensity score–matched cohort (n=398n=398) and includes PNI group, sex, race, age, fracture type, chronic pulmonary disease, white blood cell count, hemoglobin, and creatinine. Because race violated the proportional hazards assumption in the unstratified model (global test P=0.005P=0.005), race was treated as a stratification variable; the proportional hazards assumption was then satisfied (global test P=0.260P=0.260).
